# Trim24 prompts tumor progression via inducing EMT in renal cell carcinoma

**DOI:** 10.1515/med-2020-0206

**Published:** 2020-11-14

**Authors:** Tao Jiang, Houping Mao, Qin Chen, Linsheng Cao, Yanfeng He, Xingjian Gao, Wenwei Chen, Hua Zhang

**Affiliations:** Department of Urology, Second District, The First Affiliated Hospital of Fujian Medical University, Fuzhou, 350005, Fujian Province, China

**Keywords:** Trim24, renal carcinoma, epithelial–mesenchymal transition, immunohistochemistry

## Abstract

Renal cell carcinoma (RCC) is a malignant tumor originating from renal tubular epithelial cells with poor prognosis and high metastatic rate. Tripartite motif-containing 24 (Trim24) is a member of the tripartite motif (Trim) family and also a valuable oncogene, but its role in RCC remains unclear. We constructed the overexpression and knockdown of Trim24 cell lines to investigate its roles in RCC progression. CCK8, wound healing, and transwell assay were performed to determine the proliferation, migration, and invasion of RCC cell lines, respectively. Moreover, the expression of Trim24 and its clinicopathological significance were evaluated in a human RCC tissue microarray. From our results, Trim24 promoted the proliferation, migration, and invasion of RCC cells *in vitro*. Importantly, overexpression of Trim24 led to a significant increase in the expression levels of MMP-2, MMP-9, fibronectin, snail, vimentin, N-cadherin, and β-catenin, inducing the EMT process in turn, while the expression of these proteins was significantly downregulated when Trim24 was knocked down in ACHN cells. In addition, Trim24 was significantly upregulated in RCC, and its high expression was negatively associated with the tumor size. Trim24 might operate as an oncogene in RCC progression by inducing the EMT process, suggesting that Trim24 was a potential target for human RCC.

## 1 Introduction

Renal cell carcinoma (RCC) is a malignant tumor originating from renal tubular epithelial cells [1]. Approximately 30% of patients with RCC had distant metastases at the time of initial diagnosis [2]. In addition, RCC is insensitive to radiotherapy and chemotherapy [3]. The first-line treatment of RCC, interleukin-2 or interferon, has a very low rate of remission for RCC tumors [4]. All of the aforementioned reasons lead to poor prognosis of RCC patients. Therefore, the search for specific biomarkers and targeted drugs is the fundamental way to improve the survival rate of patients with RCC.

Tripartite motif-containing 24 (Trim24), initially named as transcription intermediary factor 1α (TIF1α), is a member of the tripartite motif (Trim) family and a co-regulator of the retinoic acid signaling pathway [5,6]. Recent studies reported that Trim24 is abnormally expressed in numerous tumors [7,8,9]. The increased Trim24 expression contributes to the progression of prostate cancer and is inversely related to the survival rate of breast cancer patients [10,11,12,13]. Trim24 binds to chromatin and estrogen receptors, which in turn activates estrogen-dependent genes involved in tumor cell proliferation and tumor progression [13,14]. Trim24 can promote the degradation of P53 ubiquitin, so it can be used as a therapeutic target to restore the tumor suppressor function of P53 to treat tumors [15]. In addition, Trim24 is identified as a novel dependency in acute leukemia, and Leach et al. identify Trim24 as a chemical probe of an emerging cancer dependency and establish a path forward for numerous selective yet ineffectual ligands for proteins of therapeutic interest [16]. The knockdown of Trim24 inhibits the proliferation and promotes the apoptosis in human nasopharyngeal carcinoma cells [17]. In the latest research, Trim24 impacts cell adhesion through the cross-talk with chromatin acetylation [18]. Therefore, Trim24 is a very valuable oncogene, but its expression and role in human RCC remains unclear.

Our investigation studied the expression of Trim24 and elucidated its specific role in human RCC. We offered the new evidence of cancer-promoting effects of Trim24 and developed a novel drug target for the therapy of human RCC treatment.

## 2 Materials and methods

### 2.1 Cell culture and lentivirus infection

Cells were routinely maintained in DMEM medium containing 10% fetal bovine serum (FBS) at 37°C in 5% CO_2_. Adherent cells were transferred into 24-well plates with a concentration of 1 × 10^5^ per well. After incubation for 24 h, the medium was replaced with 2 mL of DMEM medium containing 6 µg/mL polybrene, and an appropriate amount of virus suspension was added followed by incubation at 37°C for 24 h. The lentiviral vector system is Tronolab, which consists of pRsv-REV, pMDlg-pRRE, pMD2G, and transfer vector. In the present study, pLenR-GTP was used as a transfer vector. The sequence of Trim24 was inserted into pLenO-GTP plasmid through BamH I and Xba I enzyme digestion and verified by sequencing. The sequences of shRNA are as follows: Trim24-sh1, 5′ CCATGAAATGAGCCTGGCTTT 3′; Trim24-sh2, 5′ GCAGCAGTACAGCATTACTTT 3′; and Trim24-sh3, 5′ CGAGACTTATCTAAACCAGAA3′.

### 2.2 Real-time quantitative PCR

The RNA was extracted from cells by Trizol reagent (Thermo-life) and then reverse transcribed to cDNA by Go Script Reverse Transcription (Promega). The qPCR was performed by GoTaq qRCR Master Mix (Promega). The sequences of primers are as follows: Trim24 sense: 5′ AAAGGACCATCGCATGAAAC 3′, anti-sense: 5′ ATGCTGTACTGCTGCCACTG 3′. The relative quantification was determined by the 2^−∆∆Ct^ method.

### 2.3 Cell proliferation assay

CCK8 assay was performed to detect cell proliferation. After infection, cells were transferred to a 96-well plate at 100 µL/well. Fresh DMEM containing 10 µL CCK8 solutions were added. After incubation for 1 h, OD value at 450 nm was measured to determine the proliferation of cells.

### 2.4 Transwell assay

Cells (1–5 × 10^5^) were transferred in the upper transwell chamber (transwell chamber for migration experiments, transwell chambers containing Matrigel for invasion experiments). Then, 500 µL medium with 10% FBS was added to the lower chamber. Cells were blocked with 4% paraformaldehyde after incubation for 24 h and then stained with 0.1% crystal violet for 30 min. The photographs were captured by a microscope.

### 2.5 Western blotting

Cells were lysed with ice-cold RIPA buffer. About 20 µg protein sample was loaded to electrophoresis lane on SDS–PAGE gel. Then, the protein sample was transferred to PVDF membrane and blocked by using 5% fat-free milk for 1 h. The membrane was incubated with primary antibodies at 4°C overnight, followed by secondary antibody for 1 h. After washing, the membrane was applied with the ECL substrate for signal development.

### 2.6 Immunohistochemistry

RCC tissues were stained by immunohistochemistry. Rabbit anti-Trim24 polyclonal antibody was purchased from OmnimAbs. The Trim24 immunostaining score was the sum of the staining intensity and positive staining cell rate score. The staining intensity score was as follows: (0), no staining; (1), weak staining; (2), moderate staining; and (3), strong staining. The positive staining cell rate was scored as follows: (0) 0–5%; (1) 5–25%; (2) 26–50%; (3) 51–75% and (4) 76–100%. A score below 2 points was considered to be negative expression and >3 points as high expression. The score for each sample was divided into the following 4 intervals, negative (<2), + (2–3), ++ (4–5), and +++ (6–7). ≤+ was considered as negative or low expression, and ≥2+ was considered as high expression.

### 2.7 Statistical analyses

SPSS 17.0 statistical analysis software was used for the analysis of the experimental data. Immunohistochemical results were analyzed using the Pearson chi-squared test. One-way ANOVA was performed to compare the differences between the two groups. Image processing was performed using Photoshop CS3 and Adobe Illustrator CS6. *P* < 0.05 was considered statistically significant.

**Informed consent:** All the experiments in this study were approved by the local ethics committee.

## 3 Results

### 3.1 Trim24 promoted the proliferation in RCC cells

**Figure 1:**
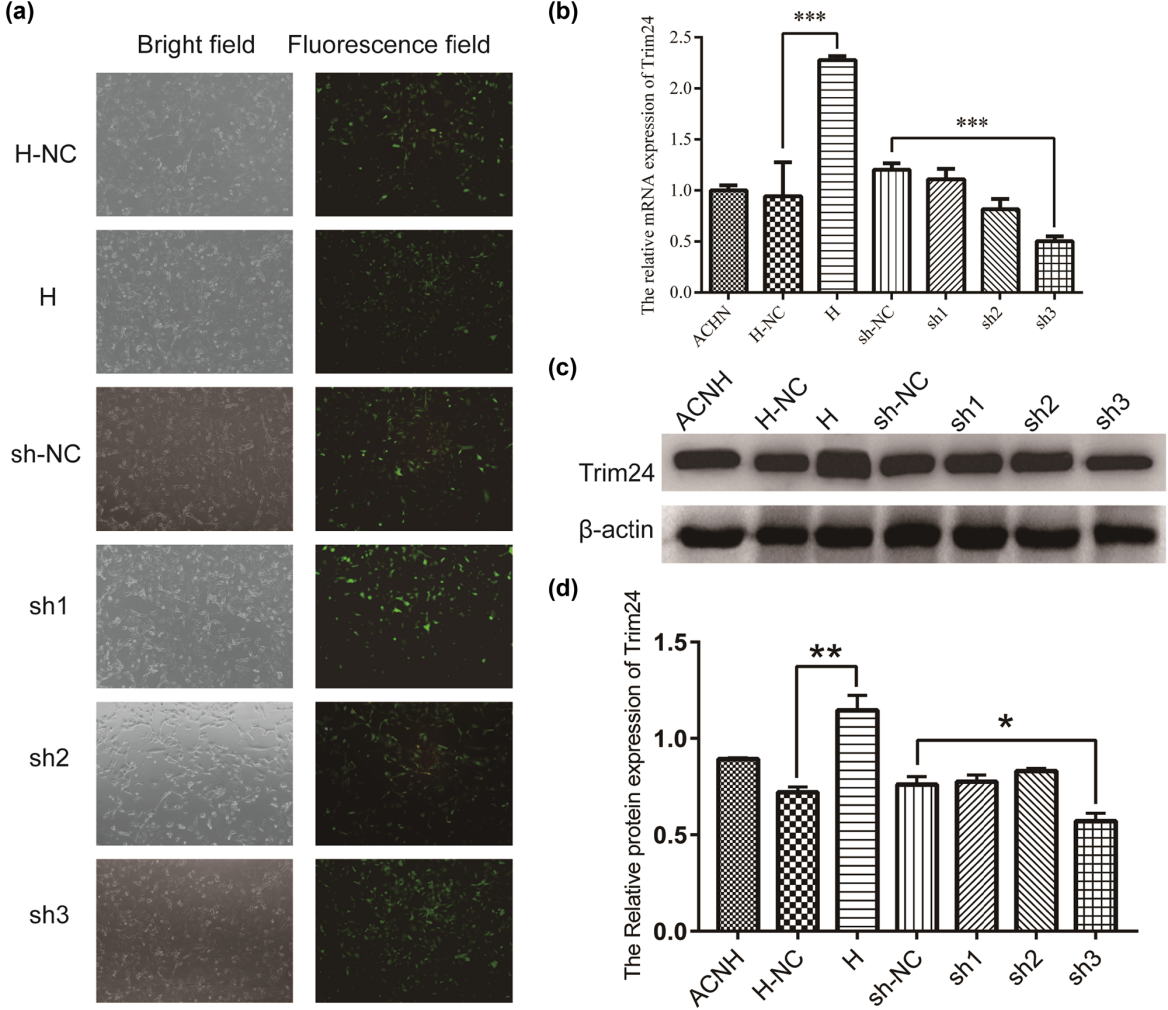
The construction of Trim24 overexpression and knockdown cell lines using lentivirus infection. ACHN cells were infected with pLenR-GTP, pLenR-GTP-Trim24, pLenR-GTP-sh-NC, pLenR-GTP-Trim24-SH1, pLenR-GTP-Trim24-SH2, and pLenR-GTP-Trim24-SH3 to generate the H-NC, H, sh-NC, sh1, sh2, and sh3 cells, respectively. (a) Bright and fluorescence intensity field at the same area were photographed using a fluorescence microscope. (b) The relative mRNA levels were detected by qPCR. (c) Western blot was performed to confirm the protein levels of these five group cells. (d) Analysis of the relative protein expression of Trim24. *P < 0.05, **P < 0.01, and ***p < 0.001.

To investigate the gene function, Trim24 overexpression (H) and knockdown (sh1, sh2, and sh3) cell lines were constructed using lentivirus infection with an empty vector as an overexpression negative control (H-NC) and a control shRNA as a knockdown negative control (sh-NC). Five transfer vector, pLenR-GTP, pLenR-GTP-Trim24, pLenR-GTP-sh-NC, pLenR-GTP-Trim24-SH1, pLenR-GTP-Trim24-SH2 and pLenR-GTP-Trim24-SH3, were build and infected ACHN cells to generate the H-NC, H, sh-NC, sh1, sh2, and sh3 cells, respectively. Fluorescence observation shows that transfection efficiency could reach more than 60% (Figure 1a). Trim24 expression was significantly increased in H in comparison to H-NC, while Trim24 expression was significantly decreased in sh3 compared with sh-NC cells (Figure 1b–d).

**Figure 2:**
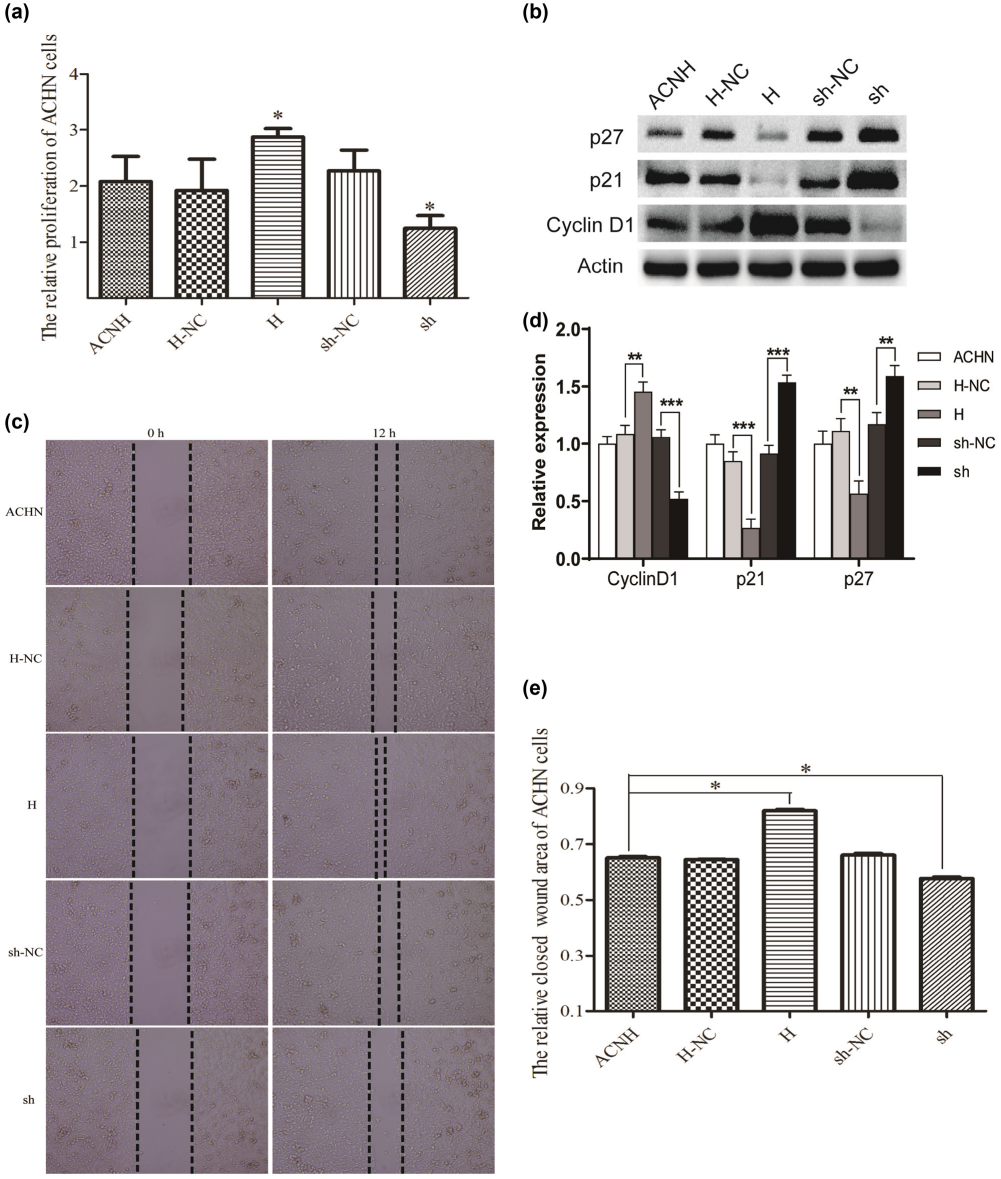
Trim24 promoted the proliferation and migration in ACNH cells. (a) Cell proliferation was estimated by the CCK8 assay. (b) The expression of p21, p27, and Cyclin D1 was detected using western bolt. (c) Gray scale was analyzed using Image J software. (d) The migration abilities were detected by wound healing assay. After transfection for 48 h, cells were photographed (100× magnification). (e) The closed wound area was analyzed using Image J software. *P < 0.05, **P < 0.01, and ***P < 0.001.

Next, we investigated the specific role of Trim24 on cell proliferation. As shown in Figure 2a, the quantity analysis of a CCK8 assay proved that the proliferation of ACNH was markedly increased in group H compared with that in group H-NC, but decreased in group sh compared with that in group sh-NC. Then, we detected the expresseion levels of cell proliferation-related proteins p21, p27, and cyclin D1 in each group by western blot. As shown in Figure 2b and c, Trim24 overexpression significantly promoted cyclin D1 expression and inhibited p21 and p27 expression, while Trim24 knockdown showed the opposite effect. Therefore, we speculated that Trim24 could promote the proliferation of RCC cells *in vitro*.

### 3.2 Trim24 promoted cell migration and invasion

**Figure 3:**
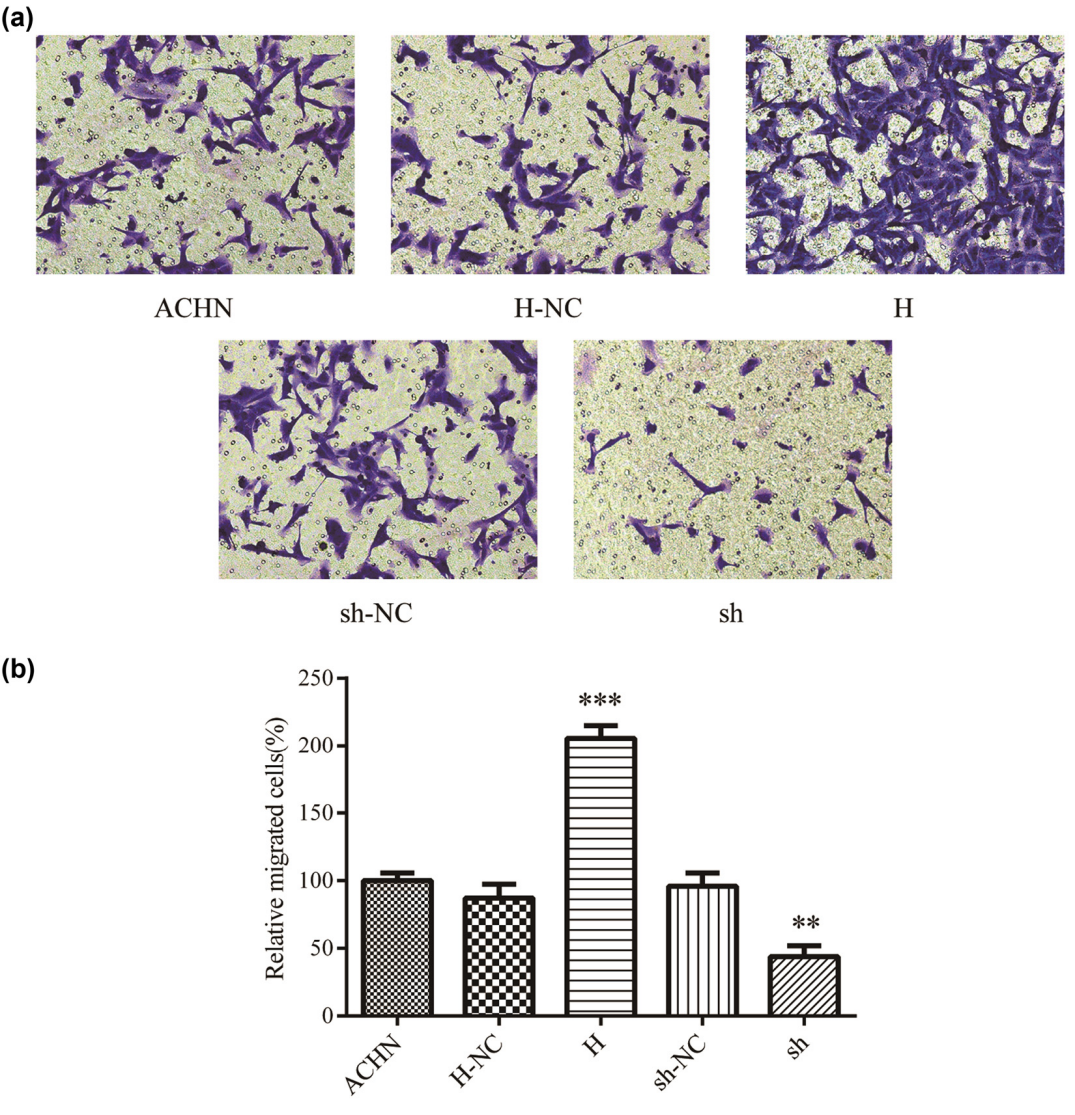
Trim24 promoted the migration of ACNH cells. (a) Transwell assay was performed to estimate the effect of Trim24 on cell migration. Migrated cells were photographed (200× magnification) after incubated in transwell chambers for 48 h. (b) Counting and analysis of migrated cell numbers. **P < 0.01 and ***P < 0.001.

**Figure 4:**
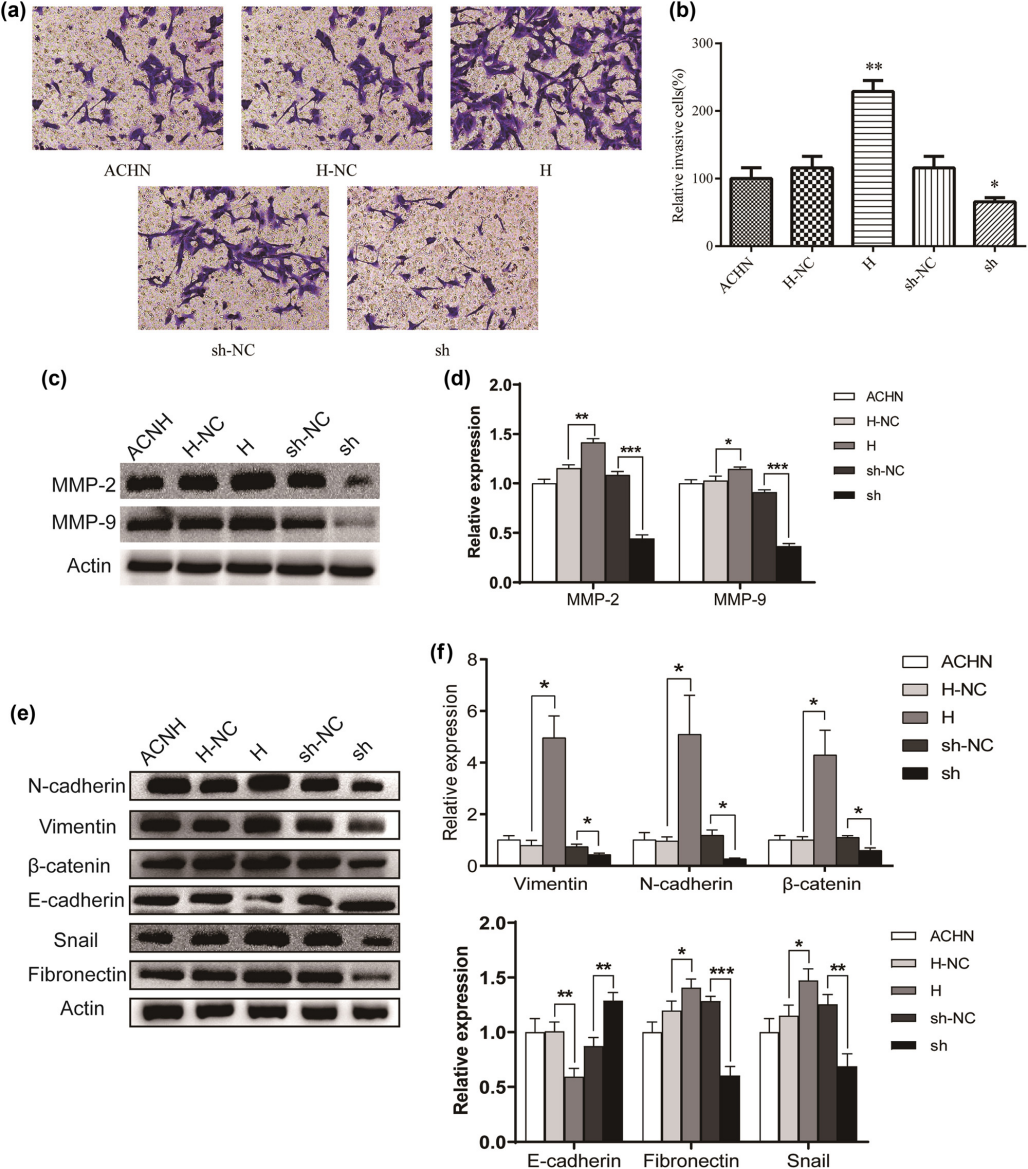
Trim24 promoted the invasion and induced epithelial-to-mesenchymal transition (EMT) process in ACNH cells. (a) Invasive cells were photographed (200× magnification) after incubated in transwell chambers with matrigel for 48 h. (b) Counting and analysis of invasion cell numbers. (c) Expressions of MMP-2 and MMP-9 were detected by the western blot. (d) Gray scale was analyzed using Image J software. (e) Expressions of key proteins in the EMT process were detected by western blot. (f) Gray scale of EMT key proteins was analyzed using Image J software. *P < 0.05, **P < 0.01, and ***P < 0.001.

The effects of Trim24 on cell migration and metastasis were evaluated using transwell and wound healing assay. The relative closed wound area of ACHN cells was significantly increased in group H compared with that in group H-NC. On the contrary, the closed wound area was significantly reduced in group sh than in group sh-NC (Figure 2d and e). To confirm the above difference, we further estimate the migratory ability of ACHN cells by the transwell assay. Consistently, the result revealed that high Tim24 expression promoted the migratory ability, while Tim24 knockdown blocked the migration of ACHN cells (Figure 3). The role of Trim24 on invasive potential in ACHN cells were also assessed using the transwell assay. Trim24 overexpression induced more cells to pass through the chambers with matrigel, while low Trim24 expression inhibited the invasive potential of ACHN cells (Figure 4a and b). Our data suggest that Trim24 promoted the migration and metastasis of RCC cells *in vitro*.

### 3.3 Trim24 induced epithelial-to-mesenchymal transition process in RCC cells

Due to the role of Trim24 on cell migration and invasion, we predict that Trim24 may be involved in the EMT process. The EMT process is associated with tumor invasion and metastasis and is characterized by the decrease of epithelial markers’ expression and the increase of mesenchymal markers’ expression. Matrix metalloproteinases (MMPs) can degrade almost all kinds of protein components in extracellular matrix (ECM), destroy the histological barrier of tumor cell invasion, and play a key role in tumor invasion and metastasis. In this research, we evaluated the EMT makers’ expressions of ACHN cells to investigate the specific effect of Trim24 on the EMT process. From our results, the expression levels of MMP-2 and MMP-9 increased significantly after the overexpression of Trim24 and decreased after the knockdown of Trim24 (Figure 4c and d). As shown in Figure 4e and f, the expressions of N-cadherin, vimentin, β-catenin, fibronectin, and snail were significantly increased in Trim24 overexpression cells (H) and reduced in Trim24 knockdown cells (sh); the expression of E-cadherin was inhibited by Trim24 overexpression and promoted by Trim24 knockdown. These results suggested that Trim24 induced the EMT process in RCC cells.

### 3.4 Expression of Trim24 in renal carcinoma and paraneoplastic tissues

**Figure 5:**
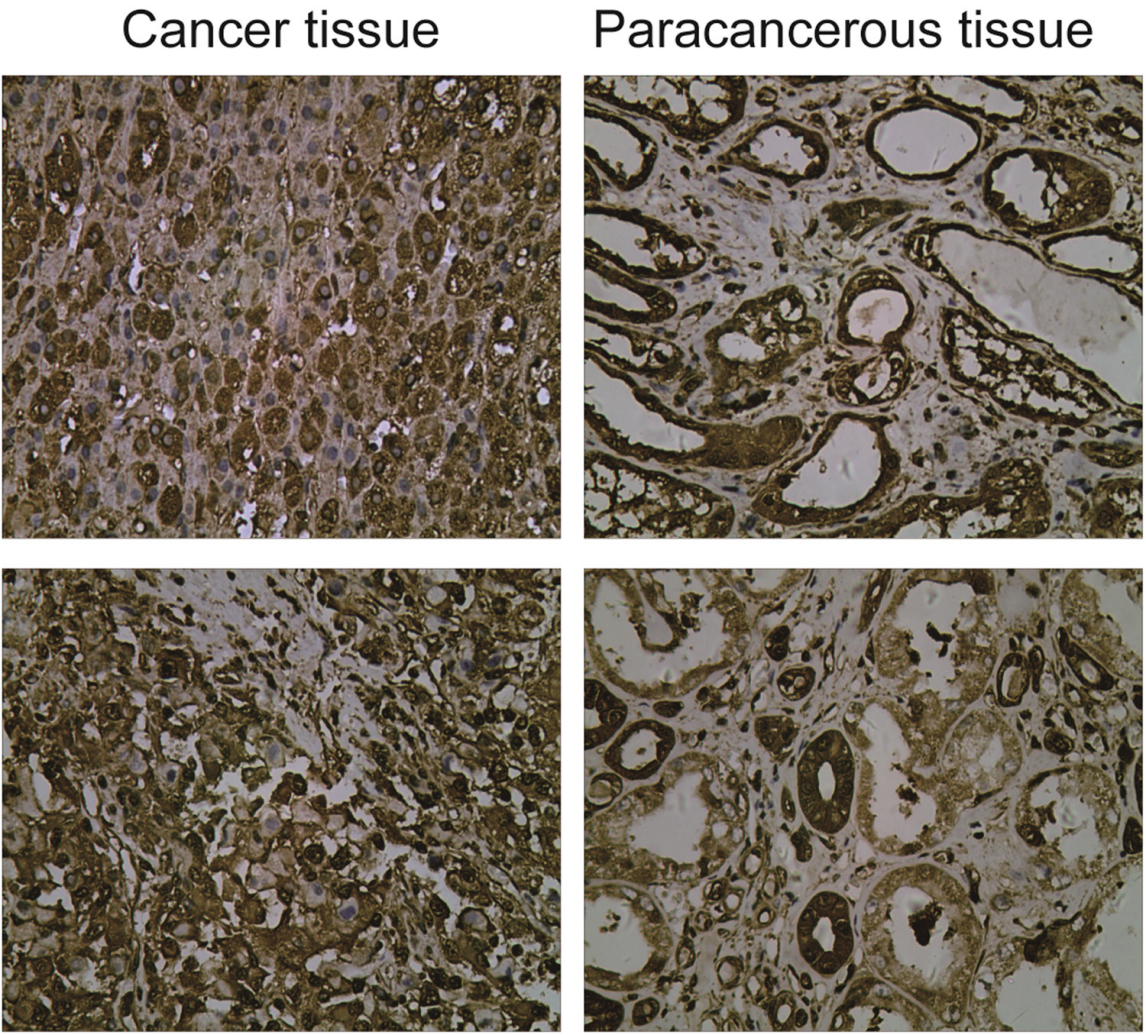
Immunohistochemical detection of Trim24 expression in renal carcinoma and paraneoplastic tissues. Representative pictures of immunohistochemistry in cancer and paraneoplastic tissues.

To acquire more intuitive understanding of endogenous Trim24 level, we performed immunohistochemical staining in RCC tissue and para-carcinoma tissue derived from the same patient. As shown in Figure 5, a strong immunohistochemistry staining of Trim24 was observed in RCC tissue, while that was very weak in normal tissue. Statistical results indicated that the high expression rate of Trim24 in tumor tissue reached 60.4% (29/48), which was markedly higher than that in paraneoplastic tissues, 27.1% (13/48) (Tables 1 and 2). In addition, we analyzed Trim24 expression in patients with different pathological parameters. Trim24 level was markedly higher in patients with a tumor diameter <5 than in those with a tumor diameter ≥5 and also higher in patients with I-II TNM stage than in those with III–IV TNM stage (Tables 3 and 4). However, Trim24 expression was not related to gender, age, and envelop infiltration and histologic grade of patients (Table 3).

**Table 1:** Statistical analysis of Trim24 expression level

	*n*	Trim24	*χ* ^2^	*P*
Renal carcinoma	48	29(60.4%)	10.84	<0.001***
Paraneoplastic tissues	48	13(27.1%)		

***P < 0.001.

**Table 2:** Relationship between Trim24 in renal carcinoma and paraneoplastic tissues

Trim24			
		*r*	*P*
Paraneoplastic tissues	Renal carcinoma	0.336	<0.001***

***P < 0.001.

**Table 3:** The relationship between Trim24 expression and pathological parameters

Pathological parameters		*n*	Trim24	*χ* ^2^	*P*
Gender	Male	35	20 (57.1%)	0.5792	0.4466
	Female	13	9 (69.2%)		
Age	<60	33	19 (57.6%)	0.3564	0.551
	≥60	15	10 (66.7%)		
Tumor diameter	<5 cm	31	14 (45.2%)	8.518	0.0035**
	≥5 cm	17	15 (88.2%)		
Envelop infiltration	Yes	28	18 (64.3%)	0.4206	0.5166
	No	20	11 (55.0%)		
Histologic grade	≤2	29	15 (51.7%)	2.315	0.1281
	>2	19	14 (73.7%)		
TNM	I–II	36	18 (27.5%)	6.534	0.0106*
	III–IV	12	11 (50.0%)		

*P < 0.05, **P < 0.01.

**Table 4:** The correlation between Trim24 expression and clinicopathological features in renal cell carcinoma

Trim24			
		*r*	*P*
Tumor diameter <5 cm	Tumor diameter ≥5 cm	0.421	0.003**
TNM I–II	TNM III–IV	0.369	0.010*

*P < 0.05, **P < 0.01.

## 4 Discussion

The Trim protein is a structurally conserved family, and more than 60 protein members of this family have been discovered in human [19,20]. Although the function of this protein family is still unclear, it has been reported that this family members may be new type of RING ubiquitin ligase, which also plays a significant role in the regulation of cell cycle and apoptosis [21]. The distinctive feature of the Trim family is its three domains, which are ring finger, B-box, coiled coil domain from the N- to C-terminus successively [22,23]. In this research, we investigated the role of an important member of Trim family, Trim24, which was an auxiliary regulator and could be combined with a retinoic acid receptor as well as a variety of nuclear receptors, such as thyroid, vitamin D3, estrogen, and Trim-mediated androgen receptors.

We constructed Trim24 overexpressing and knockout cell lines and set up controls to ensure the reliability of the experimental results. From our results, Trim24 induced cell proliferation and metastasis, which were consistent with most previous studies. Trim24 is a target gene that can generate chromosomal ectopic sites of oncogenic fusion proteins in myelodysplastic syndromes, papillary thyroid cancer, and acute promyelocytic leukemia [24,25,26]. It is also overexpressed and is positively correlated with the degree of tumor cell differentiation and P-TNM stage in nonsmall-cell lung cancer (NSCLC) [27]. Some studies have also shown that it is associated with chemoresistance in gastric cancer and glioma cells [8,28,29]. AR and Trim24 co-activated genes are found to be high expressed in prostate cancer. In addition, Trim24 bromodomain and the AR-interacting motif are fatal for cell proliferation. These data provide a theoretical basis for targeted therapy of Trim24 in speckle-type POZ protein mutant and prostate cancer patients [11].

To investigate the molecular mechanism of Trim24 in RCC, we further analyzed its relationship with the EMT process. EMT is characterized by the conversion of epithelial and mesenchymal marker molecules, which are associated with a variety of growth factors and transcription factors [30]. These transcription factors act on the promoter of E-cadherin and inhibited its transcription and expression, which is a major component of adhesion attachment and epithelial integrity markers [31]. E-cadherin, a mesenchymal marker, expression inhibition further stimulates overexpression of mesenchymal markers. Our results revealed that the expressions of MMP-2, MMP-9, N-cadherin, vimentin, fibronectin, snail, and β-catenin were significantly increased in Trim24 overexpression cells and reduced in Trim24 knockdown cells. These results suggested that Trim24 induced the EMT process in RCC cells. Moreover, Trim24 was observed overexpressed in RCC trusses according to immunohistochemically results. However, in the analysis of the relationship between clinicopathological information and Trim24 expression, the expression of Trim24 in tumor tissues with a smaller diameter was significantly higher than that with a larger diameter. These results indicated the complex role of Trim24 in tumor tissue. Recent researches prove that Trim24 plays a different role in the occurrence and the progression of multiple tumors. For example, Trim24 functions as a tumor suppressor gene in mouse liver tissue [7]. This also suggests that there is still a gap between the in vivo and in vitro environments, and further research is needed on the effect of Trim24 in RCC.

In conclusion, Trim24 promoted cell proliferation, metastasis, and induced EMT process in human RCC. Our data contributed to expanding the knowledge of Trim24 functions in promoting tumor progression, suggesting that Trim24 was a potential target for the treatment therapy of human RCC.

## Abbreviations

RCC, renal cell carcinoma
Trim24, tripartite motif-containing 24
sh-NC, shRNA negative control
